# Guided bone regeneration using a bone tissue engineering complex consisting of a poly-*dl*-lactide membrane and bone mesenchymal stem cells

**DOI:** 10.18632/oncotarget.23594

**Published:** 2017-12-22

**Authors:** Dahui Wang, Yifeng Lin, Lian Chen, Yueqiang Mo, Peng Huang, Ruixue Ma

**Affiliations:** ^1^ Department of Paediatric Orthopaedics, National Center for Children’s Health, Children’s Hospital of Fudan University, Minhang District, Shanghai 201102, China; ^2^ Institute of Pediatrics, National Center for Children’s Health, Children’s Hospital of Fudan University, Minhang District, Shanghai 201102, China; ^3^ Department of Pathology, National Center for Children’s Health, Children’s Hospital of Fudan University, Minhang District, Shanghai 201102, China

**Keywords:** developmental dysplasia of the hip (DDH), poly-dl-lactide (PDLLA) polymer, bone marrow mesenchymal stem cells (BMSCs)

## Abstract

Developmental dysplasia of the hip (DDH) is one of the most common diseases encountered in pediatric orthopedic departments. Current treatment strategies seek to improve acetabular coverage, the principal defect of acetabular dysplasia, but are not very successful. We developed a guided bone regeneration (GBR) strategy to improve acetabular coverage via bone tissue engineering (BTE). Poly-*dl*-lactide (PDLLA) membranes were seeded with bone marrow mesenchymal stem cells (BMSCs) to form a BTE complex, which was then implanted into the superior margin of the acetabulum in a rabbit DDH model. Twelve weeks later, a small amount of high-density shadowing was evident on X-rays of the superior margin of the acetabulum, specimens of which exhibited new bone formation. Micro-computed tomography yielding three-dimensional images revealed that new bone had formed in the superior acetabulum, the basal part of which had fused with (and thus reconstructed) the autogenous bone, and new trabecular bone featuring transverse interlacing was evident in the interior of the hip. No clear evidence of bone formation was observed in rabbits that underwent sham operations or that were implanted with PDLLA only. Thus, it may be possible to improve acetabular coverage via BTE-based bone regeneration.

## INTRODUCTION

Developmental dysplasia of the hip (DDH) refers to deformities of the growing hip, including frank dislocation, subluxation, instability, and dysplasia of the femoral head and acetabulum [1]. DDH is one of the most common conditions encountered in pediatric orthopedic departments, with approximately 25–50 cases per 1,000 children [1, 2]. Persistence of DDH into adolescence or adulthood may decrease knee joint strength, trigger ipsilateral genu valgum with consequent knee arthritis, cause postural scoliosis, induce a limb-length discrepancy combined with a flexion/adduction hip deformity, increase the rate of degenerative hip joint disease, and/or cause an abnormal gait [1–3]. Early diagnosis and treatment are thus critical to yield the best possible functional outcome [1–3]; a well-established correlation exists between later acetabular dysplasia and patient age at the time of reduction [1–4]. Acetabular dysplasia refers to the situation in which the superior (and usually also the anterior) femoral head is (are) incompletely covered by a dysplastic acetabulum, triggering the development of degenerative osteoarthritis [1–4]. Currently, effective treatment remains challenging [1–4].

Biodegradable polymer membranes have been widely used for guided bone regeneration (GBR) [5]. For example, poly-*dl*-lactide (PDLLA) exhibits favorable biological and mechanical properties [6]. Although biodegradable polymers offer biocompatibility, the ability to create growth space, the capacity to include cells, and clinical manageability, they cannot replace the role played by stem cells in tissue repair or regeneration [5–7]. Mesenchymal stem cells (MSCs), which are multipotent progenitor cells with the capacity to differentiate into different cell types such as chondrocytes, osteocytes, and adipocytes, have been widely used in research on tissue regeneration [7, 8]. In a partial-thickness (thus without penetration of the subchondral bone) cartilage defect model in the medial femoral condyle of the adult minipig, MSCs implanted into cartilage via intra-articular injection improved cartilage healing both histologically and morphologically [9].

Bone tissue engineering (BTE) seeks to induce functional bone regeneration employing synergistic combinations of biomaterials, cells, and growth factors [10]. BTE is a major subfield of preclinical research that is viewed as offering a potential alternative to conventional bone grafts [10]. Although combinations of MSCs and various polymers have been used in many studies of tissue regeneration, rare works have explored whether BTE might guide bone regeneration at the superior margin of the acetabulum to improve acetabular coverage [5–10]. We thus used BTE technology (a combination of MSCs with PDLLA) to explore outcomes in an animal DDH model.

In this report, homologous bone marrow MSCs (BMSCs) were isolated and seeded onto PDLLA membranes to form the BTE complex, and guided bone regeneration (GBR) afforded by this complex was evaluated in a rabbit model of DDH.

## RESULTS

### Establishment of a rabbit DDH model

**Figure 1:**
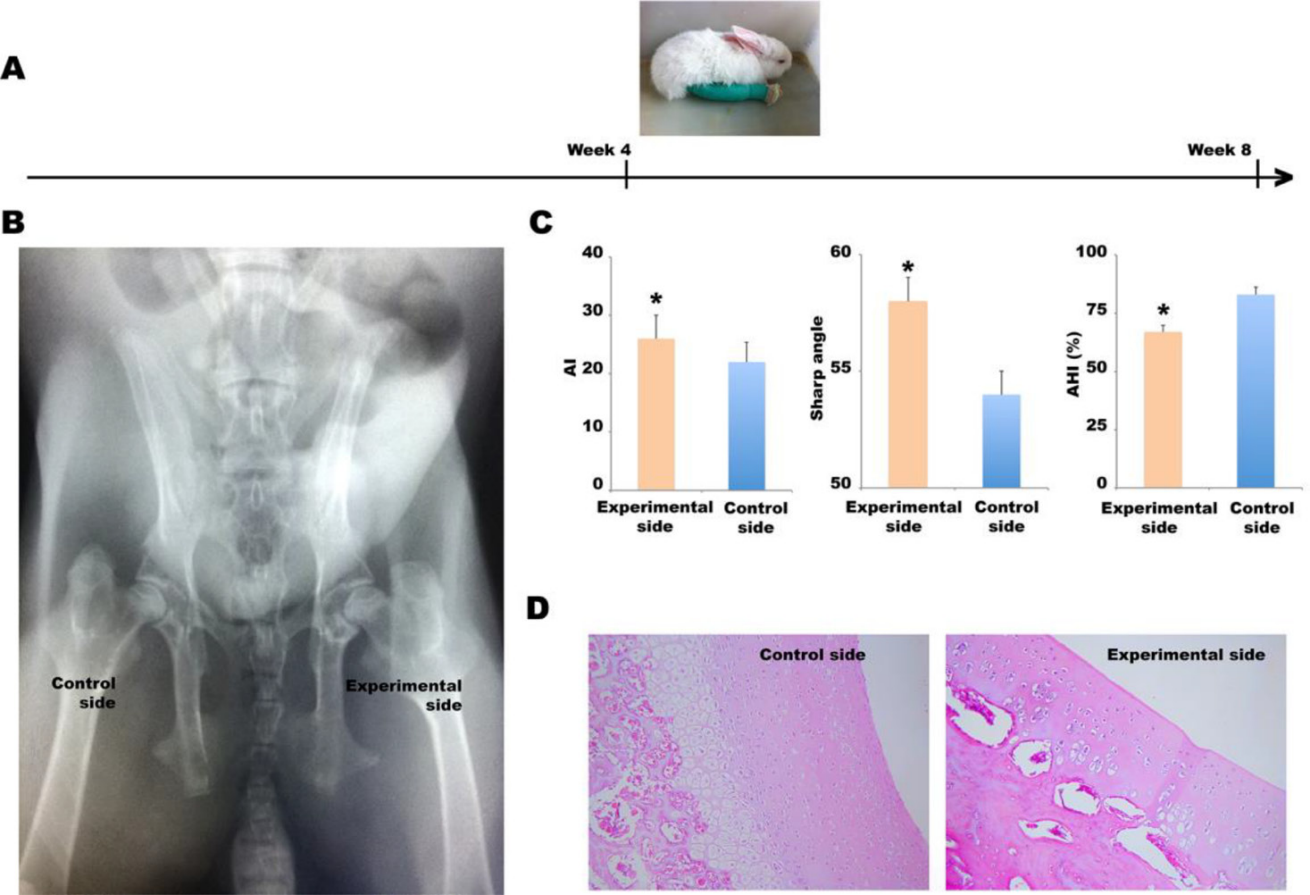
Establishment of the DDH model (**A**) The left knee joints of 4-week-old rabbits were straightened and fixed in leg cylinder casts for 4 weeks. (**B**) A posteroanterior X-ray. (**C**) The AI, Sharp’s angle, and AHI values of the experimental and control sides, ^*^P < 0.05. (**D**) Histopathological examination (hematoxylin and eosin staining) of the acetabular cartilage.

As shown in Figure 1A, the knee joints of 4-week-old rabbits were straightened and fixed with leg cylinder casts for 4 weeks. DDH was identified via hip imaging and evaluation of pathological changes. Fifteen of the 20 (75%) rabbits exhibited hip dysplasia or subluxation without dislocation (Figure 1B). The average AI and Sharp angles of the experimental sides were significantly higher than those of control sides, 26.0 ± 4.0° vs. 22.0 ± 3.4° (*P* < 0.05) and 58.0 ± 4.1° vs. 54.0 ± 3.2° (*P* < 0.05), respectively; the average AHI of experimental sides was significantly lower than that of control sides, 67.0 ± 2.9% vs. 83.0 ± 3.3% (*P* < 0.05) (Figure 1C). Thus, 75% of the rabbits exhibited pathological changes characteristic of DDH. Histopathological examination of the acetabular cartilage revealed that the DDH was associated with thin cartilage tissue, surface roughness, a disordered arrangement of middle-layer cells, and a reduction in the number of deep columnar cells (Figure 1D).

### BMSC isolation, culture, differentiation, and identification

**Figure 2:**
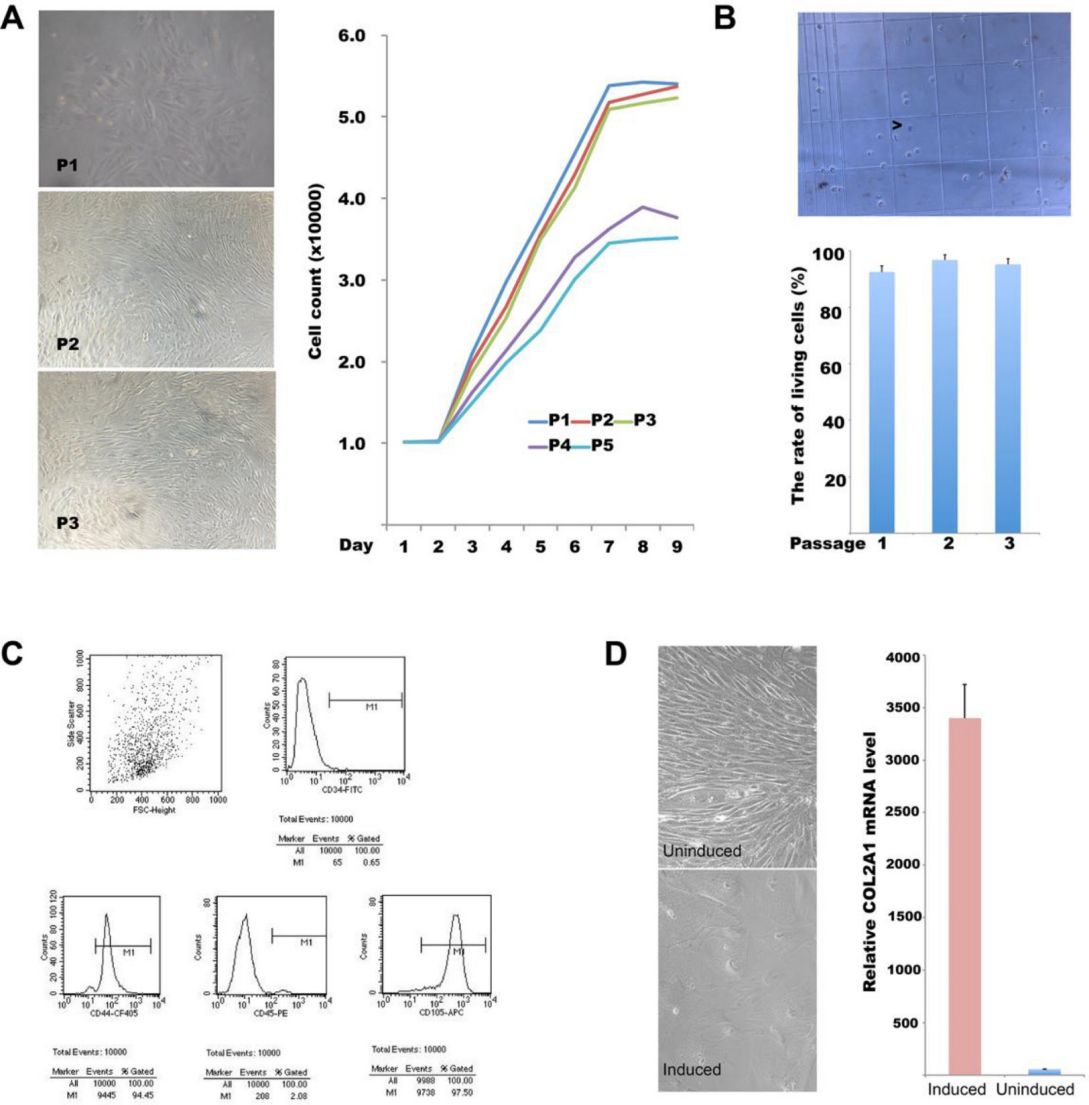
BMSC isolation, culture, differentiation, and identification (**A**) Light micrographs of BMSCs in generations 1–3 (left panel); growth curves of first- to fifth-generation cells (right panel). (**B**) Survival rates of BMSCs of generations 1–5 estimated via trypan blue staining; (**C**) CD34, CD45, CD44, and CD105 expression status on the surface of third-generation BMSCs as revealed by flow cytometry. (**D**) *In vitro*-induced differentiation of BMSCs; left panel: light micrograph; right panel: relative *COL2A1* mRNA levels in induced and non-induced cells.

BMSCs were isolated from the bone marrows of the same rabbits. After 24 h of primary culture, most cells adhered readily. In passages 1–3, the cells grew rapidly, exhibited uniform morphology and spindle-like shape, and they refracted well (Figure 2A). The growth curve showed that cell division was vigorous during the first three generations and somewhat less so over the next two generations (Figure 2A, right). In terms of viability, 92.5 ± 7.0% of primary BMSCs were viable, as evidenced by (lack of) trypan blue staining; the figures for second- and third-generation BMSCs were 96.8 ± 5.4% and 95.2 ± 4.7% respectively (Figure 2B). The average survival rate of cells of the first three generations was 94.8%. Thus, our cell isolation and culture procedure were robust; we used third-generation cells in the following experiments. These cells were exposed to antibodies and analyzed by flow cytometry (Figure 2C). Of all cells, 0.65, 94.45, 2.08, and 97.50% were positive for CD34, CD45, CD44, and CD105, respectively, suggesting that the vast majority of cells were BMSCs. Next, third-generation BMSCs at 1 × 10^8^/L were seeded into 12-well plates, and 24 h later, TGF-β1 and dexamethasone were added to final concentrations of 10 µg/L and 40 µg/L, respectively; cell morphology and *COL2A1* gene expression levels were evaluated 3 weeks later. As shown in Figure 2D, the cell volume increased, and the cells became square or triangle shaped. *COL2A1* gene expression was low prior to induction but increased by a factor of 10^3^–10^4^ after induction, suggesting that the cells had the potential to differentiate into osteogenic and cartilage cells.

### Preparation of the BTE complex

**Figure 3:**
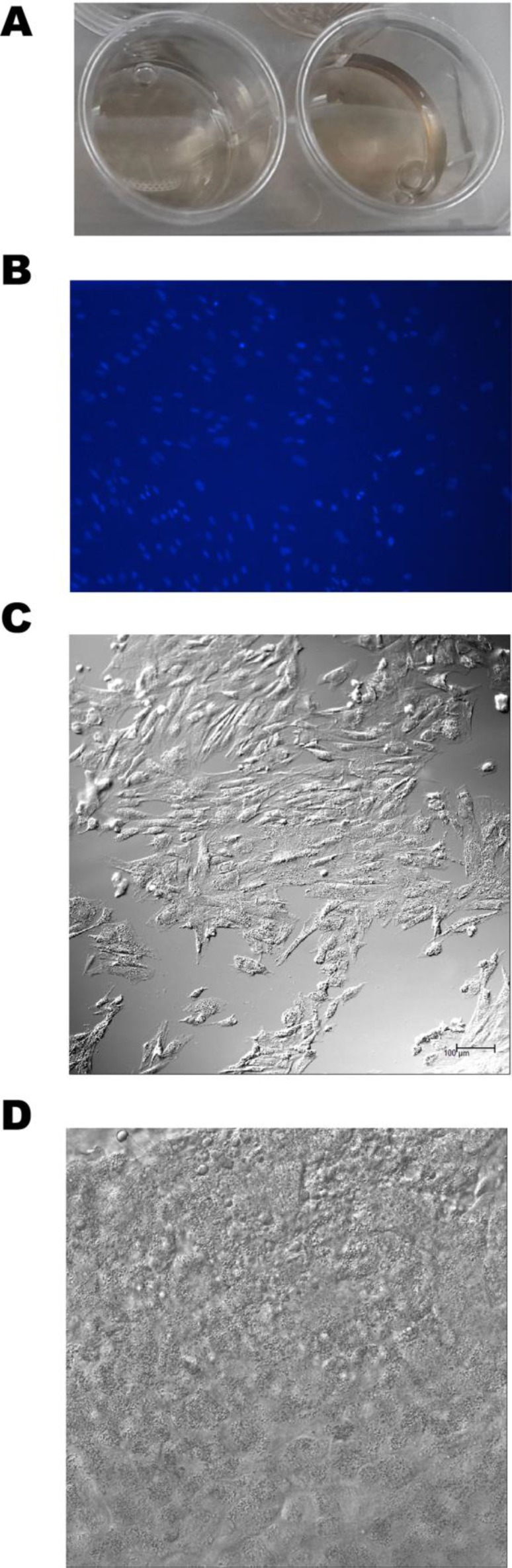
Preparation of the BTE complex (**A**) A PDLLA membrane in natural saline. (**B**) preparation of the BTE complex by co-culture of BMSCs with the PDLLA membrane; DAPI staining was checked via confocal microscopy. (**C**) A confocal micrograph confirming that BMSCs adhered to the surface of the PDLLA membrane. (**D**) A confocal micrograph confirming the spatial interaction between BMSCs and the PDLLA membrane.

The BTE complex was prepared by co-culture of BMSCs with biodegradable PDLLA membranes (Figure 3A). Proliferation of BMSCs on the membranes was confirmed by both DAPI staining and light confocal microscopy (Figure 3B–3D). After 3–5 days of culture, DAPI staining showed that the nuclei were intact and the chromatin evenly distributed, suggesting that the cells had grown vigorously (Figure 3B). Compact monolayers of spindle-shaped BMSCs were apparent on the surfaces of PDLLA membranes (Figure 3C). Under the microscope, the PDLLA membrane appeared to be porous; BMSCs adhered to the membrane surface and then fused, rendering their margins unclear (Figure 3D). Thus, the PDLLA membrane evidenced good biocompatibility.

### BTE therapy for DDH using PDLLA membranes seeded with BMSCs

**Figure 4:**
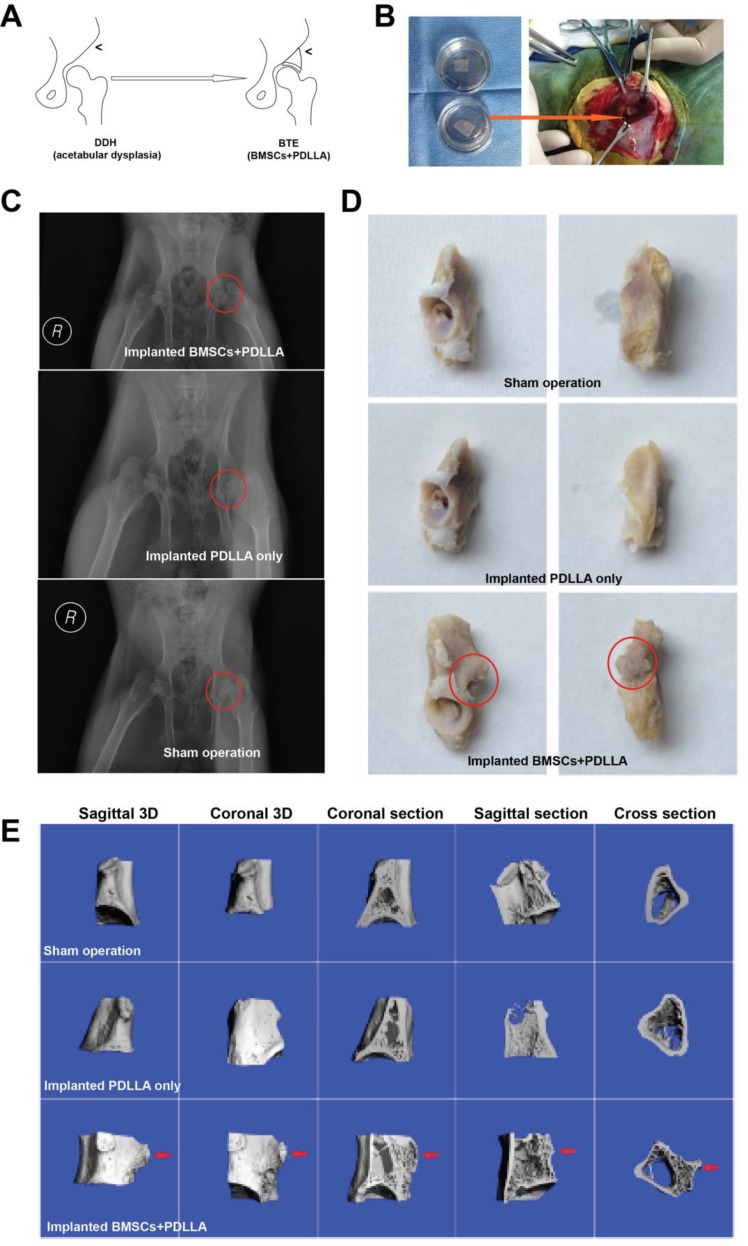
BTE-based GBR (**A**) A summary of the research flow. (**B**) A photograph of BTE-based GBR. (**C**) An X-ray taken 12 weeks after the operation; the red circle indicates sites of DDH. (**D**) Rabbit acetabular specimens; the red circle indicates new bone formation. (**E**) Micro-CT-derived 3D images confirming new bone formation.

Our goal was to improve acetabular coverage by repairing the defective superior margin of the acetabulum using BTE technology (Figure 4A and 4B). All rabbits resumed routine activities the day after the operation; no incision became infected during the 12 weeks of follow-up. Assessments were performed 12 weeks after BTE complex implantation. On X-ray examination, small high-density shadows were visible at the superior margins of acetabula implanted with BMSCs+PDLLA, but not in sham-operated animals or those who received PDLLA implants only (Figure 4C). The acetabular specimens revealed osteotylus formation in the superior margins of acetabula of rabbits implanted with BMSCs+PDLLA, but not in sham-operated animals or those who received PDLLA implants only (Figure 4D). Micro-CT and the resulting 3D images showed that new bone had formed in the superior margins of acetabula implanted with BMSCs+PDLLA, the basal portion of which had fused with (and thus reconstructed) autogenous bone, and new trabecular bone with a transverse interlacing pattern was evident in the hip interior (Figure 4E).

## DISCUSSION

DDH eventually progresses to osteoarthritis, and approximately 43% of all cases require total joint replacement in the late stages to relieve hip pain and difficulty walking [1–3, 11, 12]. Therefore, it is important to repair defects in the upper border of the hip to enhance coverage of the femoral head [1–4]. Here, we used BTE-based GBR technology to this end. First, we established a rabbit model of DDH; histopathology and medical imaging showed that 75% of rabbits subjected to hip flexion with knee extension displayed the pathological changes characteristic of DDH. Second, we isolated and cultured BMSCs of high quality, as revealed by their differentiation ability and flow cytometry. We obtained cells in quantities adequate for BTE-based GBR therapy. Third, we constructed BTE complexes by co-culture of BMSCs and PDLLA membranes; *in vitro*, we found that BMSCs used PDLLA as a scaffold on which to grow and divide, and PDLLA thus exhibited excellent biocompatibility. Fourth, GBR (implantation of the BTE complex) greatly improved the defect in the upper border of the hip. Micro-CT yielding 3D images confirmed new bone formation in the superior margin of the acetabulum. Together, our results suggest that it is possible to repair acetabular dysplasia using a BTE-based GBR approach.

Various biodegradable polymer membranes have been widely applied for GBR [5, 13]. The advantages of PDLLA include plasticity, mechanical strength, biodegradability, and a lack of association with fibrosis, adhesion, and scar development [14, 15]. *In vivo*, PDLLA is readily hydrolyzed to lactic acid, which is further converted to pyruvic acid and enters the Krebs cycle [15]. Although PDLLA resorption normally takes 6–12 months [15], 3D imaging showed that the basal part of the implant was already fused with (and thus had reconstructed) autogenous bone, and new trabecular bone with a transverse interlacing pattern was evident in the hip interior. Thus, PDLLA had supported new bone formation within 12 weeks, indicating that the surface properties, pore structure, and size of the PDLLA membrane accommodated cell proliferation by osteoblast activity of and vasculogenesis by the isolated BMSCs.

Formation of both the axial and appendicular skeletons commences early in embryogenesis via the aggregation and condensation of MSCs derived from the mesoderm germ layer [16]. BMSCs may develop into different lineages, giving rise to osteoblasts, chondrocytes, and adipocytes [7, 16]. A recent report showed that directing MSCs to bone augmented bone formation and increased bone mass [7]. However, BTE-based bone regeneration features a very complex interaction between the implanted BTE complex and the local tissue microenvironment [17, 18]. Although we have not yet defined the mechanism of bone regeneration, new bone formation employing BTE- based GBR suggests that BMSCs may trigger the expression of many growth factors and highly regulated signaling networks in a manner that is spatiotemporally appropriate.

This was primarily an exploratory study; our principal aim was to evaluate whether a combination of PDLLA and BMSCs could guide bone regeneration. We did not thoroughly evaluate the effect of BTE-based GBR on progressive dysplasia of the femoral head and the acetabulum caused by persistent DDH. We believe that improvement of acetabular coverage is key in terms of the treatment of complications attributable to persistent DDH. BTE could also be used to repair alymphatic, aneural, and avascular tissues, and to increase chondrocyte numbers in patients with DDH.

In summary, our findings suggest that it may be possible to improve acetabular coverage using BTE-based GBR. PDLLA is a good candidate biomaterial for GBR when combined with BMSCs.

## MATERIALS AND METHODS

### The rabbit model of DDH

This study was carried out in strict accordance with the recommendations of the Guide for the Care and Use of Laboratory Animals of the National Institutes of Health. The animal use protocol was reviewed and approved by the Institutional Animal Care and Use Committee (IACUC) of Fudan University.

New Zealand rabbits aged 4 weeks and weighing 0.55–0.80 kg were purchased from the Experimental Animal Department of Fudan University. DDH was established in the left hip joint by straightening and fixing the knee joints with cylindrical leg casts for 4 weeks. The contralateral extremities served as untreated controls. After the 4 weeks, pathological changes in the hip joints were evaluated on anteroposterior X-rays and via pathological examination. The acetabular index (AI, angle formed by junction of HG line with a line drawn from the acetabular surface); Sharp’s angle (the transverse angle of the acetabular inlet); and the acetabular head index (AHI, was calculated as the ratio of distance between medial tip of femoral head and lateral edge of acetabular roof to the size of femoral head) were measured. Histopathological examinations and pathoanatomical observations were also performed in our pathology department to confirm the imaging findings. Then, the rabbits were randomly assigned to three groups (five per group): a sham operation group (go through all processes as described in “Guided bone regeneration (GBR) using the BTE complex” except implantation); a PDLLA- implanted group; and a BMSC+PDLLA-implanted group.

### BMSC isolation, culture, differentiation, and identification

Local infiltration anesthesia was established at iliac bone puncture sites of 4-week-old male rabbits, using 2% (w/v) lidocaine. Autologous bone marrow (5–6 mL) was collected using 10-mL syringes containing 1-mL amounts of heparin solution (500,000 IU/L). Each bone marrow sample was labeled to ensure later autologous transplantation. BMSCs were isolated via density gradient centrifugation. Briefly, each bone marrow sample was added to a 15-mL centrifuge tube containing 3 mL of low-glucose Dulbecco’s Modified Eagle’s Medium (LG-DMEM) (Merck, Shanghai, China) and mixed gently. Then, the tube was centrifuged for 10 min at 1,000 rpm, the supernatant was discarded, and the cells were suspended in 5 mL of LG-DMEM supplemented with 10% (v/v) fetal bovine serum (FBS) (Biowest, Nuaillé, France). Separation solutions (Ficoll-Hypaque, Amersham Pharmacia Biotech, Sweden) of various specific gravities were added, and each tube was centrifuged for 25 min at 2,500 rpm. The BMSC layer (between the upper and middle layers) was transferred to another centrifuge tube and suspended in 6 mL LG-DMEM. The tube was centrifuged for 10 min at 1,000 rpm, after which the supernatant was discarded, and the BMSCs were suspended in 1 mL LG-DMEM containing 100µg/mL penicillin and 100 µg/mL streptomycin. The cells (1 × 10^6^/mL) were cultured in standard Falcon tissue culture dishes (10 cm in diameter) (Fisher Scientific, Waltham, MA) in LG-DMEM supplemented with 10% (v/v) FBS at 37° C under 5% (v/v) CO_2_. Cell growth status was checked every 24 h, and the medium was changed every 3 days; cells were passaged weekly.

BMSC quality was assessed by reference to the cell growth curve and cell survival rate, and a cell cloning assay. Briefly, cells of generations 1–5 were seeded into 24-well plates (one for each generation) at 1.0 × 10^6^ cells/well. Cells were harvested daily thereafter for enumeration of cell numbers and to allow for growth curve construction. To assess survival, 1 × 10^6^ cells in 50 µL of medium were mixed with 50 µL 0.4% (w/v) trypan blue dye for 2 min, and the living cells were counted. The cell cloning assay was performed using routine techniques.

To identify BMSCs, third-generation cells were probed with monoclonal antibodies against CD34 (FITC-conjugated mouse anti-rabbit), CD44 (CF405-conjugated goat anti-rabbit), CD45 (PE-conjugated mouse anti-rabbit), and CD105 (APC-conjugated goat anti-rabbit). All antibodies were purchased from Bio-Rad Laboratories (Shanghai, China). Cells were probed as recommended by the manufacturer and analyzed by flow cytometry on a BD FACSCanto II system (Becton Dickinson, China).

To explore the differentiation potential of isolated BMSCs, third-generation cells (1 × 10^8^/L) were seeded into 12-well plates, and 24 h later, TGF-β1 and dexamethasone were added to final concentrations of 10 µg/L and 40 µg/L, respectively. Cell morphology and collagen type II alpha 1 (*COL2A1*) gene expression were evaluated 3 weeks later. To assess *COL2A1* mRNA expression, total RNAs were extracted from 1 × 10^6^ induced and non-induced cells, and *COL2A1* mRNA levels were measured by quantitative RT-PCR. Each PCR reaction tube contained 2x One-Step Hi-Fi-PCR buffer (25 µL), total RNA (10 ng), 10 µM primer solution (1 µL), HiFi Fast DNA polymerase (2.5 U), reverse transcriptase (200 U), and water to a final volume of 50 µL. All PCR reagents were the products of Biovisualab (Shanghai, China). The PCR cycling protocol was 95° C for 2 min, followed by 35 cycles of 94° C for 20 s, 60° C for 30 s, and 72° C for 30 s. The sequences of the forward and reverse primers amplifying *GAPDH* were 5′-AGCCACATCGCTCAGACA-3′ and 5′-GCCCAATACGACCAAATCC-3′; the PCR product was 66 bp in length. The sequences of the forward and reverse primers for COL2A1 amplification were 5′-ACAG CAGGTTCACCTATACCG-3′ and 5′-CCCACTTACCG GTGTGTTTC-3′; the PCR product was 60 bp in length [11].

### The bone tissue engineering (BTE) complex

The BTE technology used in the present study included two main elements: a biodegradable PDLLA polymer membrane (Hong Jian Bio-Medical Products Co., Ltd, Guangzhou, China, http://www.hongjianbio.com) and BMSCs. Briefly, 5.0 × 4.0 × 0.4-mm membranes were rinsed in sterile normal saline three times and in LG-DMEM twice, and then laid in the bottoms of wells of six-well plates. Next, 500-µL amounts of medium containing 5 × 10^6^ third-generation BMSCs/mL were seeded into each well, and the plates were incubated at 37° C under 5% (v/v) CO_2_, with cell growth status being checked daily. After 5 days’ incubation, the BTE complexes were examined by Leica TCS SP5 confocal microscopy (Leica, Mannheim, Germany).

### Guided bone regeneration (GBR) using the BTE complex

After identification of the greater trochanter of the femur and the sciatic notch, a 6-cm-long arc incision was created in the DDH hip. Under anesthesia, the upper border of the hip joint was exposed using standard surgical procedure. Two BTE complexes (placed back to back with the cell-containing surfaces facing outward) were implanted in the superior margin of the acetabulum. The four corners were fixed with absorbable sutures, followed by continuous suturing of the muscle layer, muscle membrane, deep fascia, subcutaneous tissue, and skin. The rabbits recovered in a warm room lying on their sides with necks extended. After recovery, the rabbits were transferred to cages for further observation. X-rays were taken at 4, 8, and 12 weeks later. At 12 weeks, acetabular specimens were collected to evaluate new bone formation. Micro-computed tomography (micro-CT) (u80ScannoMedical, Zurich, Switzerland) was used to detect osteogenesis at the surgical site; three-dimensional (3D) images were obtained.

### Statistical analysis

Data are expressed as means ± standard deviations (SDs). Between-group differences were analyzed using the two-tailed t-test in SPSS version 15 software (SPSS, Inc., Chicago, IL). A *P*-value < 0.05 was considered to reflect statistical significance.
